# Relationship between cardiovascular risk factors and the echogenicity and pattern of the carotid intima-media complex in men

**DOI:** 10.1590/1516-3180.2014.1322490

**Published:** 2014-04-01

**Authors:** Priscilla Lopes da Fonseca Abrantes Sarmento, Frida Liane Plavnik, Andrea Scaciota, Joab Oliveira Lima, Robson Barbosa Miranda, Sergio Aron Ajzen

**Affiliations:** I MD, MSc, PhD. Doctoral Student of Radiology, Department of Diagnostic Imaging, Universidade Federal de São Paulo (Unifesp), São Paulo, Brazil; II MD, MSc, PhD. Affiliate Professor, Department of Nephrology, Universidade Federal de São Paulo (Unifesp), São Paulo, Brazil; III BSc. Computer Graphics Specialist, Coordinator of Teaching and Research Support for Residents and Postgraduate Students, Department of Diagnostic Imaging, Universidade Federal de São Paulo (Unifesp), São Paulo, Brazil; IV MD, PhD. Adjunct Professor, Department of Statistics, Universidade Federal da Paraíba (UFPB), João Pessoa, Paraíba, Brazil; V MD. Assistant Professor, Department of Surgery, Faculdade de Medicina do ABC (FMABC), Santo André, São Paulo, Brazil; VI MD, MSc, PhD. Titular Professor, Head of the Department of Diagnostic Imaging, Universidade Federal de São Paulo (Unifesp), São Paulo, Brazil

**Keywords:** Ultrasonography, Carotid intima-media thickness, Atherosclerosis, Risk factors, Metabolic syndrome X, Ultrassonografia, Espessura intima-média carotídea, Aterosclerose, Fatores de risco, Síndrome X metabólica

## Abstract

**CONTEXT AND OBJECTIVE:**

The thickness of the carotid intima-media complex (C-IMC) is considered to be a marker of early atherosclerosis, but visual and echogenic changes to the C-IMC can also be noted. The objective here was to evaluate the relationship between cardiovascular risk factors and the echogenicity of the C-IMC and identify those most associated with an "abnormal" C-IMC.

**DESIGN AND SETTING:**

Cross-sectional study in the ultrasound sector of the Department of Diagnostic Imaging, Universidade Federal de São Paulo.

**METHODS:**

Eighty men were evaluated. Measurements of arterial blood pressure, waist circumference (WC), lipid profile, fasting glucose, uric acid and high-sensitivity C-reactive protein were obtained. The thickness of the C-IMC was measured by means of B-mode ultrasound, and the intima-media gray-scale mean (IM-GSM) and standard deviation (IM-SD) were calculated.

**RESULTS:**

The following were discriminating variables: fasting glucose (r^2^ = 0.036; P = 0.013), uric acid (r^2^ = 0.08; P = 0.03), IM-SD (r^2^ = 0.43; P < 0.001), IM-GSM (r^2^ = 0.35; P < 0.001) and thickness of the C-IMC (r^2^ = 0.29; P < 0.001). IM-GSM showed significant correlations with WC (r = -0.22; P = 0.005), fasting glucose (r = -0.24; P = 0.002) and high-density lipoprotein cholesterol (HDL-C) (r = 0.27; P = 0.0007).

**CONCLUSION:**

IM-GSM showed correlations with WC, fasting glucose and HDL-C. However, uric acid and IM-SD presented the greatest discriminating impact. These results suggest that visual changes in C-IMC may help identify patients with potential cardiovascular risk, independently of the thickness of the C-IMC.

## Abstract (pt)

**CONTEXTO E OBJETIVO:**

A espessura do complexo íntima-média carotídeo (CIM-C) é considerada um marcador da aterosclerose precoce, mas alterações visuais e da ecogenicidade do CIM-C também podem ser observadas. O objetivo foi avaliar a relação entre os fatores de risco cardiovascular e a ecogenicidade do CIM-C e identificar aqueles mais relacionados com o CIM-C "alterado".

**TIPO DE ESTUDO E LOCAL:**

Estudo transversal no setor de ultrassonografia do Departamento de Diagnóstico por Imagem, Universidade Federal de São Paulo.

**MÉTODOS:**

Oitenta homens foram avaliados. Aferição da pressão arterial, medida da circunferência abdominal (CA), perfil lipídico, glicemia de jejum (GLI), ácido úrico (AU) e proteína C-reativa de alta sensibilidade foram obtidos. A espessura do CIM-C foi medida por ultrassom modo B e a média da escala de cinza (GSM) e do desvio padrão do CIM (DPIM) foram calculados.

**RESULTADOS:**

As variáveis discriminantes foram GLI (r^2^ = 0,036; P = 0,013), AU (r^2^ = 0,08; P = 0,03), DPIM (r^2^ = 0,43; P < 0,001), GSM (r^2^ = 0,35; P < 0,001) e espessura do CIM-C (r^2^ = 0.29; P < 0,001). Houve correlação significativa entre GSM e CA (r = -0,22; P = 0,005), GLI (r = -0,24; P = 0,002) e lipoproteína de alta densidade do colesterol (HDL-C) (r = 0,27; P = 0,0007).

**CONCLUSÃO:**

A GSM teve correlação com CA, GLI, HDL-C. Entretanto, AU e DPIM apresentaram maior impacto discriminante, sugerindo que alterações visuais do CIM-C, independentemente da espessura, podem auxiliar na identificação de pacientes com potencial risco cardiovascular.

## INTRODUCTION

Atherosclerosis is a chronic inflammatory disease that occurs mainly in large and medium-sized elastic and muscular arteries.^1^ The characteristic changes of atherosclerosis, such as inflammatory lesions or endothelial dysfunction, represent different stages of the disease.^1^

Clinically evident atherosclerosis is preceded by subclinical changes in the arterial wall.^2^ The morphological characteristics of the carotid artery, like the intima-media thickness, correspond to the existence of vascular diseases.^3^ ^,^ ^4^ Carotid intima-media thickness is a commonly used measurement of atherosclerosis,^5^ and major traditional cardiovascular risk factors, like hypertension, diabetes, obesity and hypercholesterolemia, are associated with increased carotid intima-media thickness and with the risk of cardiovascular events.^6^ Since atherosclerosis is an inflammatory disease, there is a growing amount of evidence that biomarkers like high-sensitivity C-reactive protein present increased levels in individuals with cardiovascular disease.^2^

In evaluating carotid arteries by means of ultrasound, it becomes evident from visual inspection of the intima-media complex that, even in intima-media complexes of normal thickness, there is great variation in echogenicity, texture and intima-media pattern.^5^ ^,^ ^7^ It is possible that such changes may precede development of a significant increase in the thickness of the carotid intima-media complex. 

The variations in texture and echogenicity observed by a physician during ultrasound examinations can be objectively evaluated from the visual pattern of the carotid intima-media complex and from the gray-scale mean, respectively.^5^ ^,^ ^8^ The echogenicity of the intima-media complex may also be related to risk factors other than the carotid intima-media thickness, as can be seen in the brachial artery.^5^ These changes have also been correlated with age and risk factors for atherosclerosis.^9^ In older subjects, the intima-media layer is more discontinuous and irregular, and these changes could account for the leukocytes and fatty streaks clustered along the endothelium of atherosclerotic vessels that are seen on electron micrographs.^9^ Subclinical atherosclerotic lesions typically begin as endothelial damage with gradual intima-media thickening and development of a more granular appearance on ultrasound, which represents a more advanced stage of atherosclerosis and precedes development of significant thickening.^9^

## OBJECTIVE

The objectives of this study were to evaluate the relationship between cardiovascular risk factors and the echogenicity of the carotid intima-media complex and to identify the factors most associated with recognition of an "abnormal" carotid intimamedia complex.

## METHODS

**Population**

Eighty male volunteers aged 30 to 60 years were included in this study, from March to October 2010. We used a convenience sample size to test our hypothesis in this initial study. All the volunteers were selected and referred from the nephrology outpatient clinic of Hospital do Rim e Hipertensão, Universidade Federal de São Paulo (Unifesp). They had signed a consent form, approved by the Ethics Committee of Unifesp.

The criteria for exclusion included insulin-dependent diabetes, history of smoking or use of statins. Any presence of plaque in the common carotid artery was also a criterion for exclusion.

Anthropometric measurements such as weight (kg), height (m), and waist circumference (cm) were made with the volunteer wearing light clothes and no shoes. Body mass index was calculated by dividing weight (kg) by height squared (m^2)^, and waist circumference was measured at the level of the umbilicus with the volunteers in the supine position. 

A fasting blood sample was collected in order to assess lipid profile (total cholesterol, low-density lipoprotein-cholesterol, high-density lipoprotein-cholesterol, and triglycerides), triglyceride/high-density lipoprotein-cholesterol ratio (atherogenic index), fasting glucose, C-reactive protein and uric acid.

Systolic and diastolic blood pressures were measured in the volunteers in the seated position, after five minutes of resting, using a calibrated sphygmomanometer. Three consecutive measurements were made and the average of the last two measurements was used for the analysis. 

The volunteers were divided into two groups: with and without metabolic syndrome, which was defined in accordance with the National Cholesterol Education Program/Adult Treatment Panel III (NCEP/ATP III) criteria.^10^

## Duplex scanning

The carotid intima-media thickness was measured by means of B-mode ultrasound (Accuvix V10, Medison) with a 7.5 MHz to 10 MHz linear array transducer. All the examinations were performed in the ultrasound sector of the Department of Diagnostic Imaging, Unifesp. The volunteers were examined in the supine position, by a physician who was blind to the patients' cardiovascular risk factors. Three equidistant measurements were made on the posterior wall of the right and left common carotid arteries, at a distance of 1 cm to 1.5 cm from the bulb. All the images were produced using the same time gain compensation; the depth and the B-mode gain could be altered, since these make no difference to the results.

## Data processing

**Figure 1:**
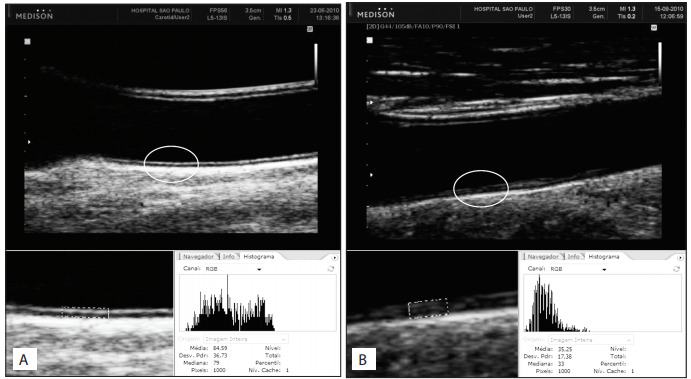
Normal double-line (A) and abnormal (B) intima-media patterns. Intima-media gray-scale mean (IM-GSM) and intima-media standard deviation (IM-SD) calculated by means of a histogram

The images were digitized and imported into Adobe Photoshop software.^9^ The region of interest was manually marked around the intima-media measurements and the program built a histogram and calculated the intima-media grayscale mean and standard deviation (Figure 1). Blood was used as a reference for black (0) and the adventitia as a reference for white (255). If the value of each white pixel was 255, one extra white pixel would be enough to modify the final intima-media grayscale mean and standard deviation values. Therefore we set the entire region of interest at exactly 1000 pixels.

## Patterns of the intima-media complex

The images were analyzed by two physicians, who were both blinded to risk factors among the patients, and the interobserver agreement was used. Based on the physicians' own experience, the arteries were classified as having a normal or an abnormal pattern. "Normal" patterns were defined as the presence of a visible double-line while "abnormal" patterns were determined by higher entropy and presence of irregularities or acoustic holes in the intima layer, or by absence of a double line.^9^ ^,^ ^11^ ^,^ ^12^ (Figure 1).

The time frame for blood sample collection, blood pressure determination, waist circumference measurement and ultrasound examination did not exceed 15 days.

## Statistical analysis

The analysis of variance test was used to evaluate differences between groups and the kappa test was used to measure the concordance between the physicians. Relationships between continuous variables were evaluated by means of Pearson's correlation coefficient and multiple regression analysis.

The data were analyzed using the Statistical Package for the Social Sciences (SPSS) 13.0 software. P-values less than 0.05 were regarded as significant.

Multiple regression analysis was used to identify the most relevant factor relating to intima-media echogenicity and the proposed model was shown to be well-adjusted to the data (adjusted r^2^ = 0.954)

## RESULTS

The average age of the study population was 49.1 ± 6.9 years. Table 1 shows the baseline data. Sixty-eight volunteers (75%) had high blood pressure and were receiving drug therapy. Nine (11.2%) of the 80 volunteers were diabetic and were using oral hypoglycemic drugs, and eight of these nine individuals also presented criteria for metabolic syndrome. Thirty-five of the volunteers (43.75%) presented metabolic syndrome and 45 (56.25%) did not have this. Only three of the 80 volunteers had no cardiovascular risk factors, although 12 of them had been considered healthy until the laboratory test results were seen.

**Table 1.** Baseline clinical dat

Variable	Without metabolic syndrome (n = 45)	With metabolic syndrome (n = 35)	P-value
Age (y)	48 ± 7	50 ± 7	> 0.05
WC (cm)	93 ± 9	107 ± 9	0.0001
SBP (mmHg)	133 ± 15	142 ± 16	0.02
DBP (mmHg)	92 ± 12	93 ± 9	0.47
HDL-C (mg/dl)	47 ± 10	37 ± 7	0.0001
TRI (mg/dl)	129 ± 65	194 ± 98	0.0007
Glucose (mg/dl)	88 ± 14	93 ± 15	0.15
Uric acid (mg/dl)	6.5 ± 1.2	7.3 ± 1.6	0.02
hs-CRP^*^	0.39 (0.02-2.79)	0.71 (0.03-6.26)	0.002
Atherogenic index	2.9 ± 1.6	5.4 ± 3.0	0.00001

WC = waist circumference

SBP = systolic blood pressure

DBP = diastolic blood pressure

**HDL-C:** = high-density lipoprotein cholesterol

TRI = triglycerides

**hs-CRP:** = high-sensitivity C-reactive protein

* value expressed as median (min-max)

Significantly higher values for waist circumference, C-reactive protein, uric acid, systolic blood pressure and triglycerides, and significantly lower values for high-density lipoprotein-cholesterol, were seen among the metabolic syndrome patients **(** Table 1 **)**.

The values for the carotid intima-media thickness were within what are considered to be the normal values on the right or left sides, but the carotid intima-media thickness was significantly higher in the individuals with metabolic syndrome (0.59 ± 0.14 versus 0.68 ± 0.16; P < 0.0007). The intima-media grayscale mean and standard deviation were different between the groups with and without metabolic syndrome, but not significantly. Table 2 shows the ultrasound analysis.

**Table 2.** Ultrasound measurement

Variable	Without metabolic syndrome (n = 90)	With metabolic syndrome (n = 70)	P-value
Intima-media thickness (mm)	0.59 ± 0.14	0.68 ± 0.16	0.0007
Intima-media gray-scale mean (IM-GSM)	38 ± 17	32 ± 15	0.08
Intima-media standard deviation (IM-SD)	17.3 ± 5.5	17.8 ± 5.3	0.09

Plaque was observed in 42 bulbs (26.25%) promoting < 50% stenosis in internal carotid arteries, which was in accordance with the criteria established by the University of Washington.^13^ No significant stenosis was measured, and there was no significant differences between the groups.

To analyze technical influences, images of the same carotid artery were produced using the same time gain compensation but changing the depth, B-mode gain and type of insonation (anterior or posterior to sternocleidomastoid muscle). No significant changes in the intima-media grayscale mean or standard deviation values were noted.

## Visual classification of the arteries

There was no significant difference between the two clinical groups in relation to the intima-media grayscale means and standard deviations, from analysis on the entire intima-media complex of 160 carotid arteries. 43.2% (69/160) of the common carotid arteries were visually classified as normal and 56.8% (91/160) as abnormal. The interobserver agreement was significant (K = 1; P = 0.0000* in the group with metabolic syndrome; and K = 0.79; P = 0.0014* in the group without metabolic syndrome).

## Correlations between 160 arteries and the clinical and ultrasound variables

In the "abnormal" arteries, the carotid intima-media thickness was greater (0.68 mm ± 0.15) than in the arteries that were considered "normal" (0.55 ± 0.11 mm) (P < 0.0001). The correlations between the carotid intima-media thickness and the variables available from the 160 arteries were significant in relation to age (r = 0.21; P = 0.007); presence of metabolic syndrome (r = 0.23; P = 0.003); fasting glucose (r = 0.20; P = 0.01); triglycerides (r = 0.39; P = 0.000); and low-density lipoprotein-cholesterol (r = 0.29; P = 0.002). 

Regarding changes in echogenicity, the intima-media gray-scale mean presented significant inverse correlations with waist circumference (r = -0.22; P = 0.005) and with fasting glucose (r = -0.24; P = 0.002); and a positive association with high-density lipoprotein-cholesterol (r = 0.27; P = 0.0007). The textural changes relating to the intima-media standard deviation only showed a significant inverse correlation with the presence of metabolic syndrome when evaluated qualitatively (Spearman's correlation: r = -0.14; P = 0.049).

Multiple linear regression analysis considering the visual classification of 160 arteries and only the clinical variables showed that the presence of diabetes *mellitus* was the only variable to have a statistically significant impact (r^2^ = 0.036; P = 0.016), in distinguishing between "normal" and "abnormal" arteries. In analyzing the laboratory variables, it became evident that increased levels of both fasting glucose (r^2^ = 0.036; P = 0.013) and uric acid (r^2^ = 0.08; P = 0.03) had significant impacts. Considering the intima-media variables, the intima-media standard deviation was the factor of greatest impact (r^2^ = 0.43; P < 0.001) followed by the intima-media grayscale mean (r^2^ = 0.35; P < 0.001) and intima-media thickness (r^2^ = 0.29; P < 0.001).

## DISCUSSION

According to the World Health Organization, there are approximately one billion people worldwide who are overweight. Of these, 300 million are obese.^14^ High serum triglyceride and low serum high-density lipoprotein-cholesterol levels and glucose abnormalities associated with obesity are also risk factors predictive of cardiovascular disease. These metabolic changes are components of the so-called metabolic syndrome.^15^ Metabolic syndrome is not clearly defined in the literature, and this definition has been continuously modified over the last few years.^10^ Despite the link between metabolic syndrome and obesity, not all obese patients have the metabolic abnormalities of metabolic syndrome. These individuals are described as "metabolically healthy obese patients".^16^ Although clinical events occur after such individuals reach their fifties, early signs of atherosclerosis can be detected in their twenties and thirties.^17^ ^,^ ^18^ The risk of future cardiovascular events and even death becomes greater with the presence of a single component of metabolic syndrome.^19^

In our study, the most prevalent cardiovascular risk factors were hypertension, obesity shown by waist circumference and elevated triglycerides. Uric acid can also be considered to be an independent risk factor for cardiovascular disease, since it causes hypertension.^20^ Increasing evidence is suggesting that uric acid may play a role in metabolic syndrome, given that hyperuricemia is present in individuals with metabolic syndrome who are not overweight or obese.^20^ We found a correlation between uric acid and intima-media echogenicity that accounted for about 8% of the change in the visual pattern.

Age was correlated with the intima-media thickness, but not with intima-media echogenicity, perhaps because of the homogeneity of the patients' ages and the small sample size for the age group.

In the overall assessment of carotid intima-media complex patterns, systolic blood pressure did not show any significant impact. One of the arguments to explain this might be the fact that all the hypertensive patients were being treated with antihypertensive medication that was not suspended at any time during the study.

Kablak-Ziembicka et al. showed that both the intima-media thickness and the C-reactive protein and tumor necrosis factor-alpha levels stratify cardiovascular events, thereby independently contributing towards the classic risk factors. Thus, these authors showed that in situations with elevated levels of these markers, the probability of event-free survival decreased.^2^ Other studies have concluded that increases in C-reactive protein levels are associated with higher cardiovascular risk only in the presence of carotid atherosclerosis.^5^ ^,^ ^7^ ^,^ ^8^ The C-reactive protein values were similar in the two groups of patients studied and showed no difference in relation to intima-media echogenicity. 

Dyslipidemia plays an important role in the progression of atherosclerosis and thickness of the intima-media complex. Statin administration has the aim of modifying the lipid profile, and the patients in this study had their medication withdrawn thirty days before the beginning of the evaluations. The ultrasound variables (intima-media gray-scale mean, intima-media standard deviation and carotid intima-media thickness) were correlated with lipid profile, such that the triglyceride levels correlated positively and significantly with carotid intima-media thickness and high-density lipoprotein-cholesterol with the intima-media grayscale mean.

Therefore, we believe that the changes in echogenicity, texture and intima-media pattern occur earlier than or occur simultaneously with the increase in intima-media thickness, given that abnormal arteries had thicker intima-media complexes, although still within the normal limits. 

Similar to what was observed with the carotid intima-media thickness, the visual changes to the intima-media complex also correlated with the presence of metabolic syndrome and with increased fasting glucose levels. Other components of metabolic syndrome such as increased waist circumference and decreased levels of high-density lipoprotein-cholesterol also correlated with an abnormal visual pattern. Thus, the artery may already be abnormal in metabolically impaired patients, but without sufficient criteria for diagnosing metabolic syndrome.

It has been suggested that the distinction between subjects with metabolic syndrome and those with metabolically impaired obesity has important implications for therapeutic medical decision-making.^19^

Patients with metabolic syndrome present greater numbers of risk factors. On the other hand, in the present study, those without metabolic syndrome also presented some risk factors like increased waist circumference and triglyceride levels. On the whole, the inflammatory and atherogenic factors are determinant in causing arterial changes and increasing the cardiovascular risk. The pattern of the arteries was significantly influenced by metabolic variables (uric acid and fasting glucose) and ultrasound variables (intima-media standard deviation).

Many investigators have regarded determining the intima-media thickness as a routine clinical practice for reclassification of cardiovascular risk in population-based studies.^21^ ^,^ ^22^ Intima-media thickness is widely used as a marker for early carotid atherosclerosis.^4^ ^,^ ^23^ Knowing the intima-media thickness is considered to improve cardiovascular risk assessments particularly among patients with intermediate Framingham scores.^22^

In type 2 diabetic patients, the extent of the carotid intima-media complex can help predict coronary events in the same way as the Framingham score, but using these two indices together significantly improves the risk prediction.^23^ Higher fasting glucose levels explained 28% of the increased thickness of the carotid intima-media complex in the patients with metabolic syndrome and about 3% of the change in echogenicity and intima-media pattern, in general. Regarding the echogenicity of the intima-media complex, its association with cardiovascular risk stratification has not yet been described.

Although the intima-media thickness in the metabolic syndrome group (0.59 ± 0.14) was significantly higher than in the non-metabolic syndrome group (0.68 ± 0.16) (P = 0.0007), the average values were within the reference limits for normal values. Regarding the changes to the intima-media pattern, some of them that were previously considered to be "normal" could be classified as either "normal" or "abnormal". Carotid arteries with low values for intima-media thickness but with changes to the intima layer should be noted in order to improve the risk stratification and medical therapy. 

Different longitudinal studies that aimed to estimate the predictive value of increased intima-media thickness have used different methods and different populations,^24^ and thus there is no consensus as to which values are the best.^25^ Perhaps the basis for defining an abnormal intima-media thickness need to be more effective:^21^ some studies have suggested that patients with values > 1.0 mm should be treated more aggressively,^21^ even including use of lipid-lowering therapies. All statins have demonstrated regression in carotid intima-media thickness.^26^ Although no study has evaluated the effect of statins on the intima-media gray-scale mean, patients using statins were excluded from the present study, or their treatment was interrupted thirty days before the evaluation.

The carotid intima-media echogenicity (intima-media gray-scale mean) has been correlated with risk factors differing from those used in relation to carotid intima-media thickness.^7^ In our opinion, although the intima-media echogenicity is less commonly used,^6^ it should also be taken into consideration.

Although numerical values for the difference between "normal" and "abnormal" artery grayscale means were not shown, lower means values for the intima-media grayscale were correlated with metabolic syndrome components such as increased waist circumference, higher blood glucose levels and lower levels of high-density lipoprotein-cholesterol, and hence with increased cardiovascular risk.

Andersson et al.^7^ showed that echogenicity was lower in subjects who had suffered a stroke than in stroke-free subjects, and that there was a non-significant trend towards a more echolucent intima-media complex in patients who had had myocardial infarction. Wohlin et al.^8^ described the intima-media grayscale mean at around the age of 50 years as a preliminary cutoff for increased mortality among elderly men. Loizou et al.^27^ observed that there was a decrease in the media layer grayscale mean with increasing age, and gave the explanation for this that in the initial stages of atherosclerotic disease, lipid concentrations increase and hyperplasia of muscle fibers occurs in the media layer, which produces hypoechoic (echolucent) structures. With aging and increasing of the media layer grayscale mean, changes to the intima-media pattern are observed; the hyperechogenic intima layer becomes less bright and the double-line pattern becomes less evident,^9^ ^,^ ^27^ or even unrecognizable.^11^ At the same time, the intima layer can present irregularities and acoustic holes.^8^ ^,^ ^9^

The intimal reflection line is included in the measurement of the intima-media grayscale mean.^5^ ^,^ ^8^ The echogenicity of this reflection line is usually related to the echogenicity of the darker space below.^5^ ^,^ ^8^

The intimal reflection line comprises only 10-20% of all the pixels in the region of interest,^5^ ^,^ ^8^ but we believe that inclusion of this line may modify the intima-media grayscale mean. For this reason, we also evaluated the intima-media standard deviation. A carotid artery with a normal double-line pattern may, when the intima layer is brighter and the media layer is hypoechogenic (darker), has the same value as a carotid without the double line and with a hyperechogenic intima-media complex. In this case, the distinction is made using the intima-media standard deviation, which is higher in carotid arteries with a double-line pattern. This suggests that the intima-media grayscale mean alone is unable to distinguish between "normal" and "abnormal" arteries. The visual criteria for normal arteries correspond to textural changes, higher entropy and higher intima-media standard deviation. Abnormal intima-media layers with atherosclerotic disease present lower values for the intima-media standard deviation.

Studies on the echogenicity of the intima-media complex have only reported isolated values for the intima-media gray-scale mean. We did not find any reports on the extent of use of the intima-media standard deviation as a cofactor in distinguishing between "normal" and "abnormal" carotid intima-media complexes with analysis on the texture of the carotid intima-media complex. According to our results, the intima-media standard deviation was the variable with the greatest impact, accounting for 45% of the change in the intima-media complex pattern.

In order to test the power of our findings, given that there is a scarcity of such data in the literature, we used the difference between the mean values for the intima-media standard deviation and the grayscale mean, comparing normal and abnormal arteries. This test made it possible to establish the sensitivity of each variable, i.e. the intima-media grayscale mean and the intima media-standard deviation.

Since waist circumference was the most significant risk factor in diagnosing metabolic syndrome, and this factor was present in 100% of such patients, and given that the echogenicity of the carotid intima-media complex was measured from the gray-scale mean, we conducted an inverse sample size calculation using Pockok's test. This test indicated the minimum sample that would be needed for this study on 69 arteries for each group (with or without metabolic syndrome). In this analysis, 90 arteries in the group without metabolic syndrome and 70 arteries in the group with metabolic syndrome were evaluated. Thus, the sample size was found to be fully coherent, adjusted and appropriate. 

The main limitation of this study was the lack of a control group. There was some difficulty in selecting healthy men between their forties and sixties. Many were considered healthy prior to clinical evaluation and laboratory tests. Even though none were diagnosed with any classical cardiovascular risk factors, it still remains questionable whether they might have had other, less prevalent factors, such as metabolic defects or genetic polymorphisms.^9^

## CONCLUSION

The classification of arteries into "normal" or "abnormal" patterns significantly influenced variables such as uric acid and metabolic syndrome components (presence of diabetes, high fasting blood glucose levels and low high-density lipoproteincholesterol levels) and the three variables of the intima-media complex (intima-media standard deviation, intima-media grayscale mean and intima-media thickness). Considering all these variables, the intima-media standard deviation was the most relevant factor. The values for the intima-media grayscale mean showed statistically significant correlations with clinically variable components of metabolic syndrome, such as high fasting glucose levels, low high-density lipoproteincholesterol and increased waist circumference. The intimamedia standard deviation had a significant correlation with the presence of metabolic syndrome.

Prospective studies are necessary in order to confirm the importance of these results in relation to normal values for the intima-media thickness and evaluate whether these changes have any clinical impact on predictions of cardiovascular events.
